# Tissue heterogeneity is prevalent in gene expression studies

**DOI:** 10.1093/nargab/lqab077

**Published:** 2021-09-03

**Authors:** Gregor Sturm, Markus List, Jitao David Zhang

**Affiliations:** Biocenter, Institute of Bioinformatics, Medical University of Innsbruck, 6020 Innsbruck, Austria; Pharma Research and Early Development, Pharmaceutical Sciences, Roche Innovation Center Basel, F. Hoffmann-La Roche Ltd, Grenzacherstrasse 124, 4070 Basel, Switzerland; Chair of Experimental Bioinformatics, TUM School of Life Sciences Weihenstephan, Technical University of Munich, 85354 Freising, Germany; Pharma Research and Early Development, Pharmaceutical Sciences, Roche Innovation Center Basel, F. Hoffmann-La Roche Ltd, Grenzacherstrasse 124, 4070 Basel, Switzerland

## Abstract

Lack of reproducibility in gene expression studies is a serious issue being actively addressed by the biomedical research community. Besides established factors such as batch effects and incorrect sample annotations, we recently reported *tissue heterogeneity*, a consequence of unintended profiling of cells of other origins than the tissue of interest, as a source of variance. Although tissue heterogeneity exacerbates irreproducibility, its prevalence in gene expression data remains unknown. Here, we systematically analyse 2 667 publicly available gene expression datasets covering 76 576 samples. Using two independent data compendia and a reproducible, open-source software pipeline, we find a prevalence of tissue heterogeneity in gene expression data that affects between 1 and 40% of the samples, depending on the tissue type. We discover both cases of severe heterogeneity, which may be caused by mistakes in annotation or sample handling, and cases of moderate heterogeneity, which are likely caused by tissue infiltration or sample contamination. Our analysis establishes tissue heterogeneity as a widespread phenomenon in publicly available gene expression datasets, which constitutes an important source of variance that should not be ignored. Consequently, we advocate the application of quality-control methods such as *BioQC* to detect tissue heterogeneity prior to mining or analysing gene expression data.

## INTRODUCTION

The genome-research community has witnessed the exponential growth of gene expression studies in the last two decades, first with microarray (1) and nowadays with RNA-seq datasets (2). Both the huge volume of data and wide coverage of biological samples in diverse contexts, such as genetic perturbation, disease progression, pharmaceutical intervention, *etc*. make publicly available gene expression studies an important resource for biomedical research. Systematic mining of existing data and interrogation of new data can reveal molecular foundations of pathology and disease (3), identify novel therapeutic targets (4), enable preclinical screening tools for drug safety (5,6), highlight mode-of-action of drug candidates (7), allow data-driven prioritization of drug screening hits (8), and enrich and stratify patients as well as predict their response to therapeutics (9). In short, gene expression studies are indispensable for both disease understanding and drug discovery in biomedical research.

However, the power of gene expression studies in translating molecular biology into medicine is impeded by a lack of reproducibility (10,11). Well-known causes of irreproducibility include batch effects, lack of annotation, variation of biological samples, profiling protocols or data analysis procedures, mistakes in sample handling or annotation, and in rare cases intentional data manipulation. Several studies have scrutinized publicly available gene expression datasets and demonstrated the prevalence of impact by these factors, especially batch effects (12) and sample misannotation, which is reported to affect at least one-third of samples even if only the donor sex label is considered (13). In contrast, the community has yet to assess the prevalence of *tissue heterogeneity*, i.e. the unintended profiling of cells of other origins than the tissue of interest (14,15). Tissue heterogeneity can be caused by intrinsic characteristics of the sample to be profiled, such as the tumour microenvironment or immune cell infiltration into solid organs, or by extrinsic factors such as imperfect dissection or contamination of samples. Ignoring tissue heterogeneity reduces statistical power of data analysis and can, in the worst case, invalidate the conclusions of a study. In particular in oncology, this is a well recognized problem that is commonly addressed by estimating tumour purity (16). On the other hand, cell type heterogeneity can be leveraged as a source of information in immune cell deconvolution to inform about the state of the tumour microenvironment and to guide immunotherapy (17). Beyond tumour samples, Nieuwenhuis *et al.* identified a cluster of pancreas-specific genes that were expressed in tissues other than pancreas not only in GTEx but also in other datasets, highlighting that tissue contamination is an important issue affecting important reference datasets commonly used by the community (15).

While both the causes and consequences of tissue heterogeneity have been established, its prevalence in public gene expression data remains unknown. A systematic analysis of tissue heterogeneity with respect to cross-tissue contamination is missing. The outcome of such an analysis would both benefit retrospective data analysis and integration efforts as well as inform the design of analysis protocols of gene expression data generated in the future. To fill this critical gap, we systematically study two large public gene expression repositories, Gene Expression Omnibus (GEO) (18) and ARCHS4 (19), using the previously reported R package *BioQC*, and a reproducible, open-source Snakemake (20) workflow employing a new Python package *pygenesig* developed for this study. Focusing on a subset of nine tissues with rigorously validated gene expression signatures and 2 667 studies that fulfilled a set of stringent filtering criteria, we find that tissue-heterogeneity is widespread, affecting at least 5.8% samples. The prevalence varies by tissue type in both microarray and RNA-seq datasets independently of the time when the study was deposited in the public domain. Our results urge all researchers dealing with gene expression studies to consider tissue heterogeneity as a confounder in data analysis and to take actions to reduce or avoid its impact on reproducibility.

## MATERIALS AND METHODS

### Compilation and cross-validation of tissue signatures

**Figure 1.**
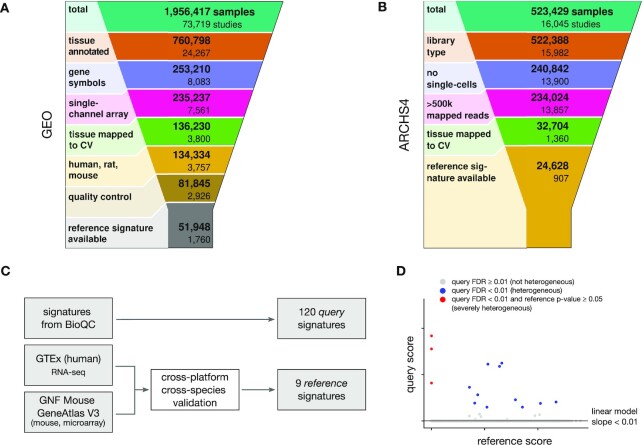
(**A** and **B**) Selection of gene expression studies from (A) GEO and (B) ARCHS4. (**C**) We defined two sets of tissue signatures for the analysis: (i) we obtained 120 tissue *query* signatures from the *BioQC* package and (ii) generated 9 high-quality *reference* signatures from the GTEx and GNF Mouse GeneAtlas V3 datasets. (**D**) Schematic illustration of our approach to detecting heterogeneous samples. Since query signatures may be imperfect and correlated with the sample’s tissue of origin, we excluded signatures that were correlated with the reference signature (robust linear model slope ≥ 0.01). We defined a sample as *heterogeneous*, if a query-signature was detected at an FDR < 0.01. We define a sample as *severely heterogeneous* if additionally, the reference signature was not detected at a *P*-value of 0.05. Abbreviations: CV, controlled vocabulary; FDR: false-discovery rate.

*BioQC* provides 155 sets of tissue-enriched genes (tissue signatures hereafter) derived from four large-scale tissue gene expression datasets (14). Even though the authors have shown that the signatures are biologically meaningful, they did not validate them using an independent dataset. Since the reliability of signatures is crucial for this study, we developed an open-source software package, *pygenesig*, which facilitates the creation and validation of tissue signatures. We applied *pygenesig* to transcriptomics data from the GTEx project (21) (v6) which contains 11 984 samples from 32 tissues and validated the resulting signatures on the GNF Mouse Gene Atlas V3 (22). We identified a set of nine reference tissue signatures that reliably identify their tissue of origin, regardless of experimental platform and species after rigorous validation. The process of signature generation and validation is outlined in Figure 1C and detailed in Section S2 of Supplementary Data.

### Gene expression data corpus

We retrieved annotation and gene expression data from GEO on 7 December 2016 using *GEOmetadb* (23) and *GEOquery* (24). We downloaded consistently processed RNA-seq gene expression data including annotations as binary RData objects from the ARCHS4 project website (19) on 10 February 2020 (version 8.0). Data filtering and quality control are summarized in Figure 1A,B and described in detail in Section S3 of Supplementary Data.

Tissue annotations in GEO and ARCHS4 are inconsistent. Therefore, we manually mapped tissue descriptions to a controlled vocabulary, thereby assigning 120 of the 155 signatures provided by *BioQC* and the nine reference signatures to their corresponding tissues (Supplementary Table S1).

### Detecting tissue heterogeneity with BioQC in the corpus

*BioQC* performs a Wilcoxon–Mann–Whitney statistical test for enrichment of a certain signature on a per-sample basis. We ran *BioQC* on all samples from GEO and ARCHS4 using the 9 reference signatures and 120 signatures provided by *BioQC*, which yielded 9 878 304 (sample, signature, *P*-value) pairs. As signatures can be correlated (e.g. because they describe developmentally or physiologically related tissues), we exclude correlated signature pairs so that they do not inflate false-discovery proportions.

**Figure 2.**
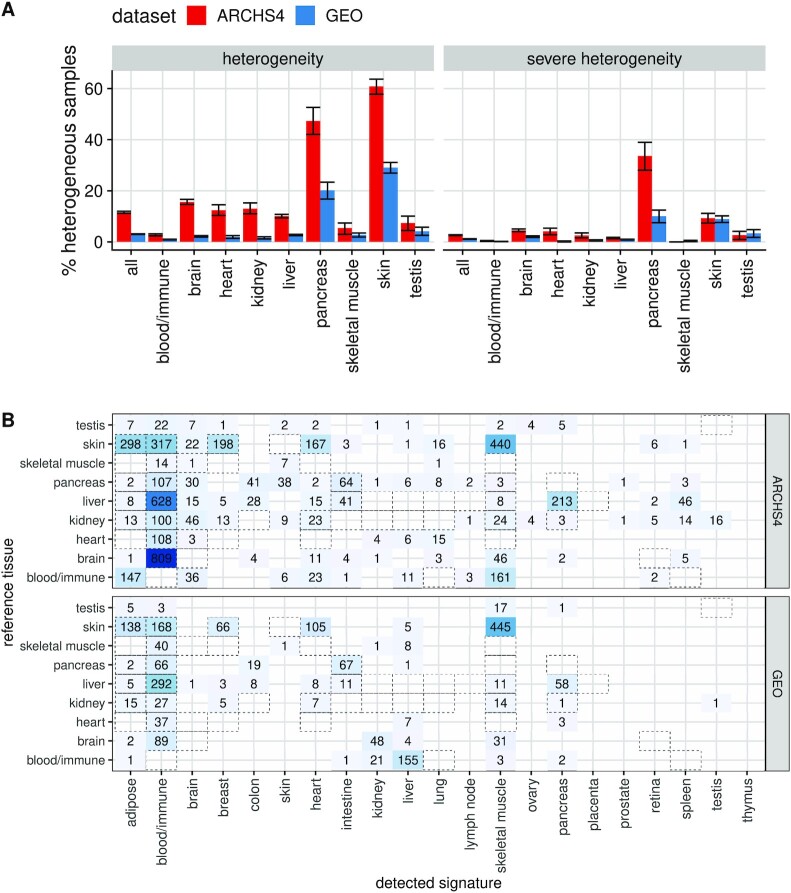
Tissue heterogeneity in gene expression studies from GEO and ARCHS4. (**A**) Fraction of heterogeneous samples per tissue. Error bars show 95% confidence intervals derived by bootstrapping (*n*=1 000). (**B**) Confusion matrix of tissues with absolute counts. Reference tissue refers to the annotated tissue, detected signature to other tissue signatures that were detected in these samples by *BioQC*. For tiles boxed with dashed lines, one or more query signatures have been removed due to correlation with the reference signature.

The detection process consists of five steps as illustrated in Figure 1D and in Section S4 of Supplementary Data. A given sample *s* annotated as tissue *t* is tested for enrichment with the query-signature *k*_query_ resulting in a *P*-value *p*_query_. Let *k*_ref_ be the reference signature associated with tissue *t* and *p*_ref_ the *P*-value of testing *s* for enrichment of *k*_ref_. Let $\tau$ be the false-discovery rate (FDR) threshold. (i) If the Benjamini–Hochberg (BH)-adjusted *p*_query_$ \ge \tau,$we label *s* as not heterogeneous, else continue. (ii) We fit a robust linear model using rlm from the R package *MASS* of |log_10_(*p*_query_)| against |log_10_(*p*_ref_)| for all samples annotated as *t*. (iii) If the slope of the linear model is ≥ 0.01, we exclude the pair of signatures from the results. If the slope is < 0.01 and the FDR-adjusted *p*_query_ < $\tau$, we consider the sample as heterogeneous. Tissue pairs for which signatures are excluded are marked as such in Figure 2B. (iv) We define heterogeneity as *severe*, if additionally the unadjusted *p*_ref_ > 0.05. (v) Finally, we compute the fraction of heterogeneous samples by dividing the number of samples that have at least one signature passing the above criteria by the total number of samples per tissue. Confidence intervals have been derived by bootstrapping (*n*=1 000) using the R package *boot*.

### Documentation and the pipeline

We implemented and documented the analysis using the R package *bookdown* (25). The analysis is wrapped into a reproducible pipeline built on *Snakemake* (20).

## RESULTS

We designed and implemented an analysis workflow to estimate the prevalence of tissue heterogeneity in publicly available gene expression datasets. We evaluate the enrichment of 120 *query signatures* from the R package *BioQC* in a selection of well annotated gene expression studies in the GEO (18) and ARCHS4 (19) repositories (2 667 studies, 76 576 samples, Figure 1A,B). These query signatures are tissue-sensitive, i.e. they recognize their target tissue with few false negatives but often not tissue-specific, i.e. they report false positives due to the expression of the signature genes in other, physiologically similar tissues. To account for this, we propose a set of nine *reference signatures* using GTEx data (21) and validate them using the GNF MouseAtlas V3 dataset (22) to show that they are robust even across species (Figure 1C). For each tissue, we exclude all query signatures that are correlated with the reference signature and consider a sample heterogeneous if one of the remaining signatures is detected at an FDR < 0.01 (Figure 1D). We further distinguish between severe and moderate tissue heterogeneity. Empirically, we define moderate heterogeneity as samples that are significantly enriched for a signature that we do not expect to be present, and severe heterogeneity as samples in which, in addition, the expected signature of the annotated tissue is not detected. While severe heterogeneity often suggests mistakes in sample handling and annotation, moderate heterogeneity suggests contamination or infiltration with blood or immune cells.

We find moderate tissue heterogeneity in about 5.8% of all samples and severe heterogeneity in 1.6% of samples. The proportion of samples affected by moderate heterogeneity varies by the organ and tissue being profiled, with skin (40%) and pancreas (30%) samples affected most frequently and blood samples affected least frequently (1.4%) (Figure 2A), which intuitively agrees with the complexity of the respective sampling procedures.

In general, heterogeneity was higher in ARCHS4 than in GEO, which can likely be attributed to the higher sensitivity of sequencing compared to microarrays. However, the overall patterns (highest heterogeneity in pancreas and skin) are comparable between the platforms, suggesting that the issue of sample heterogeneity is platform-independent. We further observe that heterogeneity is not equally distributed across studies. While most studies (84.3% in GEO and 73.8% in ARCHS4) contain no samples with detected heterogeneity, a considerable proportion (5.9% GEO, 7.3% ARCHS4) contains ‘severely heterogeneous’ samples (Supplemental Figure S8). Using a linear model, we conclude that tissue heterogeneity is not associated with the year of the study, suggesting that this issue exists since the early days of transcriptome profiling and persists (Supplementary Figure S9).

A closer investigation of the source of tissue heterogeneity reveals additional insights (Figure 2B). First, enrichment of blood signatures in other tissues and organs is one of the most frequent forms of severe heterogeneity. Multiple causes are possible: it can be caused by an increased inflow and/or decreased outflow of blood which sums as a net increase of blood volume, or the activation and proliferation of tissue-resident leukocytes, for instance. Besides heterogeneity related with blood, many instances of tissue heterogeneity are caused by proximal tissues, which could be explained by imperfect separation of nearby organs. For example, the liver and pancreas are proximal organs connected by the common bile duct, which may explain why many cases of tissue heterogeneity in pancreatic tissue are caused by liver-specific tissue signatures. Finally, tissue heterogeneity involving distal solid tissues also occurs, which may indicate contamination during sample preparation. Considering that the latter two aspects represent technical biases, such samples should be excluded in analysis to increase statistical robustness and to avoid arriving at erroneous conclusions.

## DISCUSSIONS AND CONCLUSIONS

Due to a lack of annotation, only a small fraction of GEO microarray datasets (12.9%) could be used for our analysis. In particular a lack of tissue annotation disqualified the majority (61%) of the datasets. Since *BioQC* depends on the ranking of genes within a sample, per-gene normalized expression profiles in the GEO repository could also not be evaluated. We find it especially problematic that no standardized mapping from probe id to gene symbols was available for many of the remaining samples. The low percentage of usable samples begs the question if our findings generalize to the entire sample population in GEO. However, many of the trends we observed in GEO (e.g. that skin and pancreas exhibit the highest level of heterogeneity) are also found in ARCHS4, which collects data of different samples acquired with an entirely different technological platform.

We also note that the issue of tissue heterogeneity is specific to bulk gene expression data and does not affect single-cell RNA-seq studies, as contaminating cells form an independent cluster of cells which can either be ignored or incorporated in data analysis. In fact, single-cell RNA-seq offers the chance to study biological sources of tissue heterogeneity at a previously unimaginable depth. However, due to its lower cost and simpler sampling procedure, the majority of expression profiles will still be sequenced in bulk in the foreseeable future. In addition, many studies strive to use information derived from bulk-sequenced samples to inform both experimental design and analysis of single-cell studies. Hence, identifying samples affected by tissue heterogeneity will remain an important aspect of data analysis. Standard methods, such as principal component analysis (PCA) can identify heterogeneous samples as outliers. Using signature-based methods such as *BioQC* has the additional benefit of explaining the source of variance, and even works with single samples, or when all samples are affected.

A limitation of the study is that we focused only on bulk gene expression datasets based on mRNA profiling using either microarray or Illumina sequencing. High-throughput gene expression data derived from other modalities, such as third-generation sequencing techniques and mass spectrometry-based proteomics, usually require many cells as input and hence may suffer from tissue heterogeneity as well. A recent study by Yoo *et al.* (26), for instance, reported a community effort to address sample mislabelling issues in proteogenomic and multi-omics studies, and found 7.5% and 3.5% mislabelled samples in two datasets. To our best knowledge, tissue heterogeneity has not been addressed on a large scale by such a community effort. Future studies are warranted to explore the landscape of tissue heterogeneity in data generated from alternative gene-expression profiling techniques.

Detecting and modelling tissue heterogeneity is of particular importance in systems medicine studies, where tissue-specific signals can mask disease-specific signals, thus preventing the successful detection of disease mechanisms, patient stratification, or drug target identification and validation. Based on the prevalence of tissue heterogeneity in gene expression data, we advocate the routine use of methods such as *BioQC* to assess tissue heterogeneity and to ensure reproducibility of gene expression studies.

## DATA AVAILABILITY

The reference signatures and raw results table including all accession numbers tested is available from https://doi.org/10.5281/zenodo.4298774. The source code to reproduce the analysis can be found at https://github.com/grst/bioqc_geo. *Pygenesig* is available from https://github.com/grst/pygenesig. *BioQC* is available from R/Bioconductor and the documentation is available at https://accio.github.io/BioQC/.

## Supplementary Material

lqab077_Supplemental_Files
